# Acoustic metamaterial for subwavelength edge detection

**DOI:** 10.1038/ncomms9037

**Published:** 2015-08-25

**Authors:** Miguel Molerón, Chiara Daraio

**Affiliations:** 1 Department of Mechanical and Process Engineering (D-MAVT), Swiss Federal Institute of Technology (ETH), 8092 Zurich, Switzerland; 2 Division of Engineering and Applied Science, California Institute of Technology, Pasadena, California 91125, USA

## Abstract

Metamaterials have demonstrated the possibility to produce super-resolved images by restoring propagative and evanescent waves. However, for efficient information transfer, for example, in compressed sensing, it is often desirable to visualize only the fast spatial variations of the wave field (carried by evanescent waves), as the one created by edges or small details. Image processing edge detection algorithms perform such operation, but they add time and complexity to the imaging process. Here we present an acoustic metamaterial that transmits only components of the acoustic field that are approximately equal to or smaller than the operating wavelength. The metamaterial converts evanescent waves into propagative waves exciting trapped resonances, and it uses periodicity to attenuate the propagative components. This approach achieves resolutions ∼5 times smaller than the operating wavelength and makes it possible to visualize independently edges aligned along different directions.

## Abstract (web summary)

Super-resolution imaging is based on the restoration of evanescent and propagative waves. Here, the authors present an acoustic metamaterial that transmits only components of the acoustic field equal to or smaller than the operating wavelength, which can be used to provide sharp images of the edge of an object.

## Graphical abstract

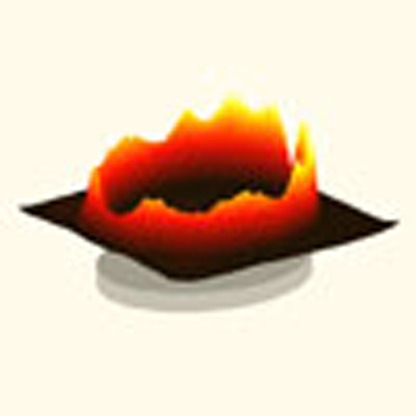

Edge detection is an essential numerical tool in image processing that finds application in several areas of science and technology. In medical imaging^1^,^2^, non-destructive testing^3^,^4^ and computer vision^5^, edge detection plays an important role, since it enables extracting the meaningful information from an image, and it reduces the amount of data to be processed. The basic idea behind this technique is to high-pass filter the image to remove the low spatial frequencies. Close to the edges of an object illuminated by a monochromatic wave, the wave field is dominated by evanescent waves, that is, waves with spatial oscillations faster than the operating wavelength. A device capable of generating an image using only evanescent waves would visualize the edges or small details of an object, essentially extracting only the key information contained in the image.

There exist different ways to detect evanescent waves that overcome the classical diffraction limit of conventional imaging devices. Approaches based on superlenses^6^,^7^,^8^,^9^,^10^,^11^,^12^,^13^,^14^,^15^,^16^,^17^ and hyperlenses^18^,^19^ or time reversal techniques^20^,^21^,^22^,^23^ allow restoring evanescent waves, providing a detailed picture of the imaged scene. However, since propagative waves that carry low spatial frequencies are also used to form the image, such techniques cannot be used to visualize only fast wave field variations.

Here we report a metamaterial design that provides an image of only spatial variations of the acoustic field that are equal to, or smaller than, the operating wavelength. This approach provides sharp images of the edge of an object, with resolution up to *λ*/7.6 (*λ* is the operating wavelength). Moreover, the technique allows visualizing edges aligned along a given direction independently.

## Results

### Metamaterial transmission properties

**Figure 1:**
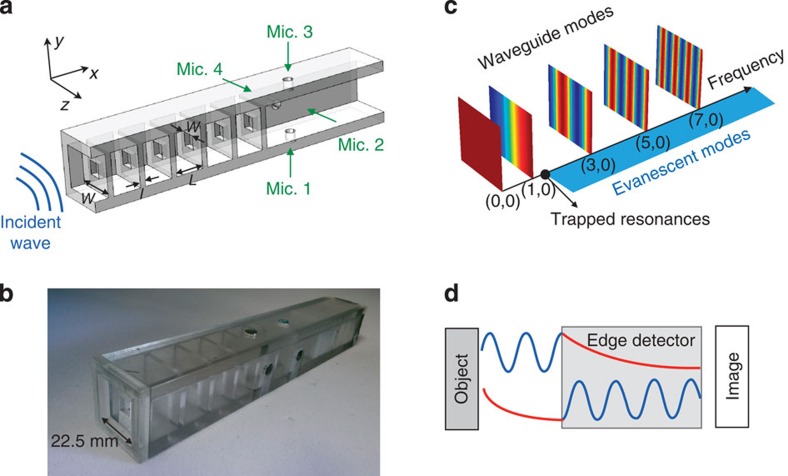
Imaging device. (**a**) Illustration of the metamaterial consisting of a periodic array of five symmetrical resonators. The frontal wall is not shown in order to expose the internal structure. The holes pointed by green arrows indicate the microphones position in the experimental study. (**b**) Experimental realization using 3D printing. (**c**) Eigenfunctions of modes (0,0), (1,0), (3,0), (5,0) and (7,0), and their position in the frequency axis. At the TR frequencies (black dot) only modes (0,0) and (1,0) are propagative. (**d**) Basic illustration of the operating principle of the device.

The proposed metamaterial consists of a wave-guiding structure with an array of coupled symmetric resonators, as shown in Fig. 1a,b. The sample has two different square cross-sections: a narrow section, *s*, and a wide section, *S*, with dimensions *s=w* × *w* and *S*=*W* × *W*. The axial lengths of the narrow and wide segments are denoted, respectively, by *l* and *L*. The symmetric variations of the cross-section generate trapped resonances (TRs), which appear at frequencies slightly above the cutoff frequency of the first antisymmetric mode in the wide section^24^,^25^, *f*=*c*_0_/2*W*, where *c*_0_=343 m s^−1^ is the sound speed. More details about the origin of these resonances are given in the Supplementary Note 1 and Supplementary Fig. 1. These resonances are antisymmetric with respect to the longitudinal axis (Supplementary Fig. 2), meaning that they can only be excited by antisymmetric-guided modes (Fig. 1c shows some of these modes). The excitation of the TRs induces a strong coupling between high-order antisymmetric modes, including the evanescent ones, making it possible to tunnel subwavelength information through the device. On the other hand, the periodicity induces a bandgap for the plane mode, which avoids the transmission of components with small perpendicular wavenumber. By choosing the geometrical parameters, it is possible to make the plane mode bandgap and the TRs coincide in frequency, creating a spectral band in which only waves with large perpendicular wavenumber are transmitted. The frequency of the TRs and the position and width of the plane mode bandgap can be adjusted, respectively, by *W*, the period in the *x*-direction, *l*+*L* and the ratio *w*/*W*. The coincidence of the TRs with the plane mode bandgap is achieved for a wide range of values of *w*, *W*, *l* and *L*. In the particular case considered in this work, these parameters are *w*=7.5 mm, *W*=22.5 mm, *l*=3 mm and *L*=15 mm.

Figure 1d illustrates the principle of operation of the device: low spatial frequencies, carried by propagative waves (blue sinusoidal lines), are converted into evanescent waves (red decaying lines). High spatial frequencies, carried by evanescent waves, are converted into propagative waves. As a result, a picture of only the edges of the imaged object is created. Note that there is a fundamental difference between this principle of operation and that of previous subwavelength imaging approaches^7^, which also restore propagative components. These features arise from the fact that the plane mode excitation is not involved in the restoration of the evanescent waves, unlike previous approaches using acoustic resonance^8^,^9^,^10^,^11^,^12^,^13^,^14^,^15^,^16^,^26^.

Using the multimodal method (Supplementary Note 2), we have calculated the transmission matrix **T**, **a**^t^=**T a**^i^, where vectors **a**^i^ and **a**^t^ contain the incident and transmitted modal amplitudes. The transverse eigenmodes, *φ*_(*m,n*)_(*y*, *z*) are denoted by the couple (*m*, *n*), indicating the number of nodal lines parallel to *y* (subscript *m*) and *z* (subscript *n*), with *m* and *n* as integer numbers. The terms of the transmission matrix are denoted by 
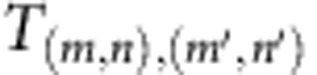
, indicating the coupling of the transmitted mode (*m,n*) with the incident mode 
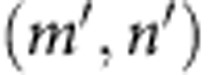
.

**Figure 2:**
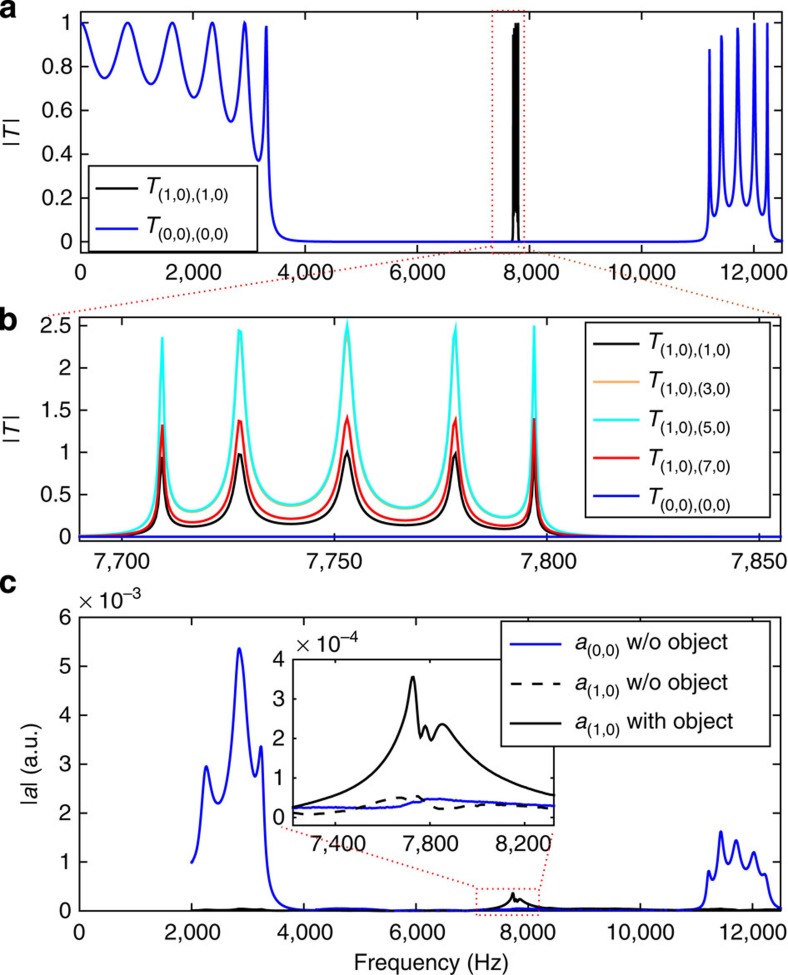
Transmission properties. (**a**) Modulus of the transmission coefficients *T*_(0,0)_,_(0,0)_ (blue line) and *T*_(1,0),(1,0)_ (black line). The propagating band ∼7.75 kHz is due to the excitation of the TRs. (**b**) Zoom-in of the band containing the TRs. (**c**) Measured transmitted amplitudes of modes (0,0) and (1,0). The blue solid line and the black dashed line represent, respectively, the amplitudes of modes (0,0) and (1,0), measured without object. The black solid line represents the amplitude of mode (1,0) when an object is placed close to the input section.

Figure 2a shows the plane mode transmission coefficient, *T*_(0,0),(0,0)_, and the term corresponding to the first antisymmetric mode, *T*_(1,0)(1,0)_, in the frequency band (0,12.5) kHz. The curve corresponding to mode (0,0) shows a wide bandgap between ∼3.7 and 11 kHz. This bandgap is created by the strong scattering of the plane mode in the metamaterial, owing to the large contrast between *w* and *W*. In the absence of TRs, the transmission coefficients of any higher-order mode should be also equal to zero in the frequency band studied, as the cutoff frequency of the first higher-order mode in the narrow section is *f*=*c*_0_/2*w*=22.8 kHz. However, we see a propagative band in the transmission term *T*_(1,0),(1,0)_ appearing at frequencies slightly above the cutoff frequency of the first antisymmetric mode in the wide section, *f*=*c*_0_/2*W*=7,622 Hz, generated by the TRs (Supplementary Note 1). Figure 2b zooms in the frequency band containing these resonances. We observe five transmission peaks between 7,700 and 7,800 Hz, corresponding to the transmission of mode (1,0) with amplitude equal to one. The five peaks are due to the evanescent coupling between the five identical resonators forming the metamaterial. In this figure, we also show the terms *T*_(1,0),(3,0)_, *T*_(1,0)(5,0)_ and *T*_(1,0),(7,0)_. Note that modes (3,0), (5,0) and (7,0) are all evanescent at these frequencies. However, Fig. 2b puts in evidence the ability of the device to convert evanescent waves into propagative ones. Remarkably, we observe conversion of modes (3,0), (5,0) and (7,0) with amplitudes >1, demonstrating the possibility to transfer efficiently subwavelength information through the device. Identical results are obtained for *z*-antisymmetric modes (0,1), (0,3), (0,5) and (0,7).

The coupling of the incident field *p*^*i*^(*y,z*) with the eigenmodes *φ*_(*m,n*)_(*y,z*) is given by:


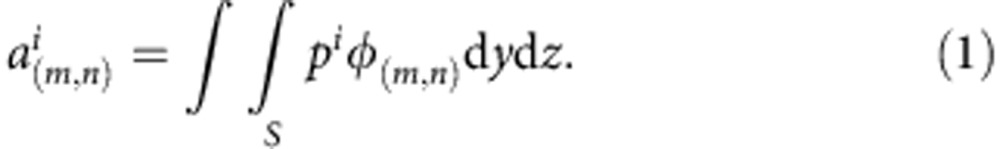


Equation (1) indicates that slow spatial oscillations couple mainly with the plane mode, which cannot propagate at the TRs' frequencies. In contrast, fast spatial asymmetric oscillations couple with the high-order antisymmetric modes and are transmitted through the device. However, since for any antisymmetric mode, equation (1) vanishes if *p*^*i*^ is symmetric, symmetric excitations cannot be transmitted.

To test these ideas experimentally, we measure the transmitted amplitudes of the plane mode, *a*_(0,0)_, and the first two antisymmetric modes *a*_(0,1)_ and *a*_(1,0)_. A loudspeaker was placed on the longitudinal axis 280 mm away from the metamaterial input section. The transmitted amplitudes of modes (0,0), blue solid line, and (1,0), black dashed line, are shown in Fig. 2c. The plane mode amplitude exhibits a bandgap in the band (3.7,11) kHz, as predicted in Fig. 2a. Since the acoustic source is placed symmetrically with respect to the longitudinal axis, none of the antisymmetric modes are excited, and therefore the amplitude of mode (1,0) is close to zero in the whole frequency range. This situation changes when an object is placed close to the input section. The black solid line in Fig. 2c represents the amplitude of mode (1,0), measured when the edge of an aluminium plate was placed at ∼1 mm distance from the metamaterial, blocking half of the input aperture. In this configuration, the rapid variations of the acoustic field around the edge couple with the antisymmetric modes (equation (1)), which in turn excite the TRs and transmit signals around the TRs' frequencies (see the inset of Fig. 2c). In the experiments, we distinguish only three transmission peaks (instead of five, as predicted theoretically in Fig. 2b), and attribute this to inherent losses, not taken into account in our model.

Using our theoretical model, we found that the amplitude of the transmission peaks in Fig. 2b is independent of the number of unit cells in the metamaterial. Thus, since the resolution depends on the conversion amplitude of evanescent modes into propagative modes, the performance is in principle independent of the metamaterial length. However, our model does not take into account the losses in the metamaterial. When losses are taken into account, one should expect the amplitude of the transmission peaks to decrease with the length, degrading the image quality. From this constraint, it is convenient to build a metamaterial with a reduced number of unit cells. On the other hand, the larger the number of unit cells, the strongest the attenuation of the plane mode, which improves the separation of the evanescent field from the propagative field. We found that five unit cells provided a good trade-off between strong plane mode attenuation and high conversion amplitude of evanescent modes.

### Edge visualization

**Figure 3:**
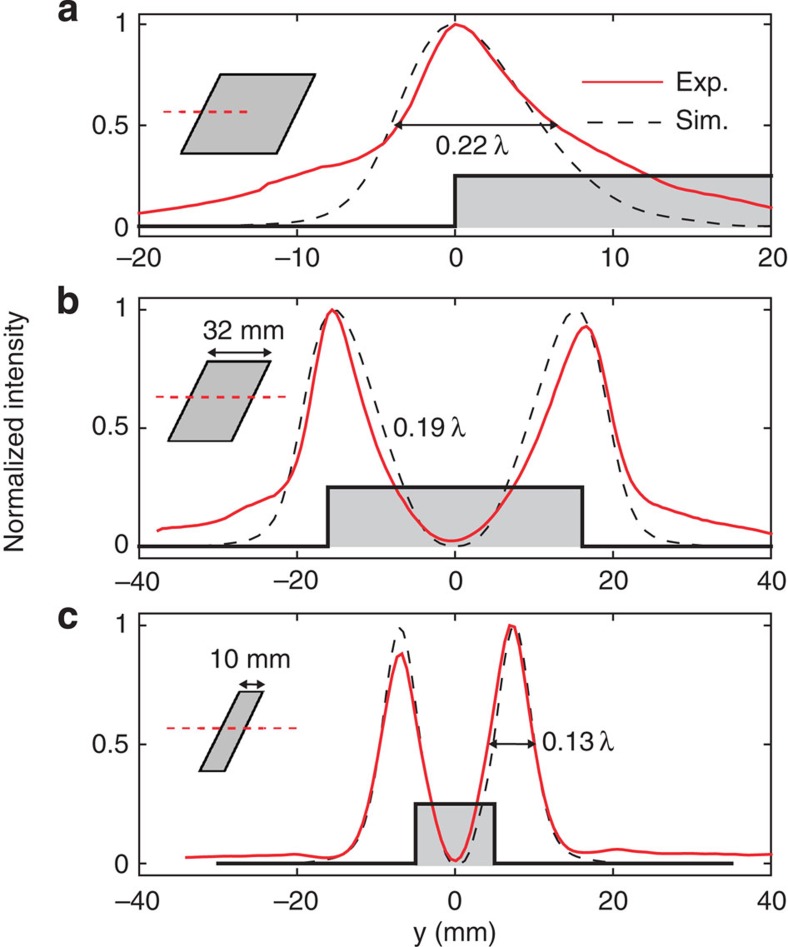
1D Scans. Images of (**a**) an edge of an aluminium plate, (**b**) the two edges of a 32-mm-wide aluminium plate and (**c**) the two edges of 10-mm-wide aluminium rod. The insets show the imaged objects, in which the dashed red line represents the scanned region.

We performed a series of experiments to image the edges of different objects. Figure 3a shows a one-dimensional (1D) scan of the edge of an aluminium plate, the edges of a 32-mm-wide aluminium plate are shown in Fig. 3b, and the edges of a 10-mm-wide aluminium rod are shown in Fig. 3c. The frequency chosen is *f*=7,740 Hz (*λ*=44.3 mm), corresponding to the maximum of *a*_(1,0)_ in Fig. 2c. The experimental results were compared with finite elements simulations performed with Comsol Multiphysics (see more details in the Methods section). Solid red lines and dashed black lines represent, respectively, experimental and numerical results. The single edge (Fig. 3a) generates a sharp peak in the transmitted intensity of mode (1,0). The resolution, defined as the full-width at half-maximum (FWHM) of the peak, is 0.22*λ*. The two edges of the 32-mm plate are seen as two narrow peaks with FWHM=0.19*λ* (Fig. 3b). A special situation arises when imaging small objects, whose edges are separated by a distance close to the resolution obtained for a single edge (0.22*λ*≈10 mm). Remarkably, the device still generates two sharp peaks with FWHM=0.13*λ* (Fig. 3c). However, the resulting image does not represent the actual object size, but an object slightly larger. The reason for this is that when the position of the object is symmetrical with respect to the longitudinal axis (*y*=0), the transmitted intensity drops to zero, which shrinks the peaks generated by the edges.

**Figure 4:**
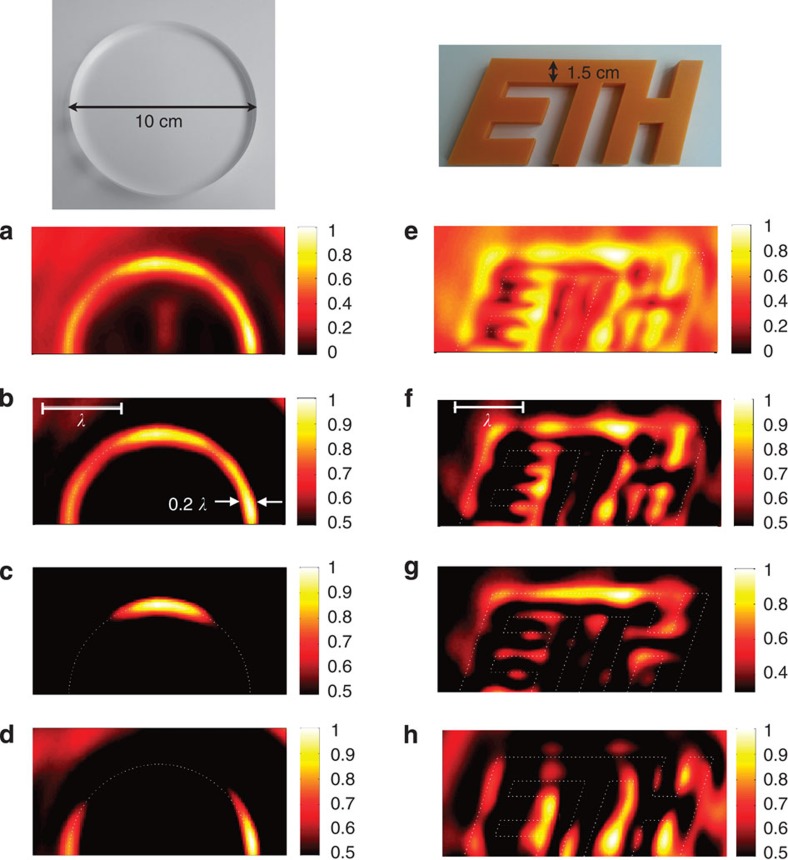
2D Scans. (**a**–**d**) Images of a 10-cm-diameter disc. (**e**–**h**) Images of the ETH Zurich logo. A picture of these objects is displayed above these figures. (**a**,**e**) The normalized total intensity, *I*=*I*_(1,0)_+*I*_(0,1)_. (**b**,**f**) The total intensity but limiting the dynamic range to (0.5,1). (**c**,**g**) *I*_(1,0)_, which enables to visualize only horizontal edges. (**d**,**h**) *I*_(0,1)_, which enables to visualize only vertical edges.

We have tested experimentally device's ability to image two-dimensional (2D) objects. In particular, we have imaged a 10-cm-diameter plexiglas disc and the ETH Zurich logo, made of a rigid thermoplastic (Fig. 4). Figure 4a shows the total transmitted intensity *I*=*I*_(0,1)_+*I*_(1,0)_, when imaging the plexiglas disc (only the upper half disc is represented). The intensity is maximum at the edges of the disc (represented by the dotted line). The image also shows other features with lower amplitude, that can be generated by unwanted reflections in the experimental setup. A clearer image is obtained by reducing the dynamic range to one half of the maximum intensity (Fig. 4b). A semicircle is clearly observed in the image, with FWHM≈0.2*λ*. As mentioned above, an interesting aspect of this technique is the possibility to visualize edges aligned along different directions. This is achieved by visualizing the intensities *I*_(1,0)_ (for horizontal edges) or *I*_(0,1)_ (for vertical edges) separately. Such separation is enabled by the choice of the microphones' positions, which makes it possible to measure independently modes (1,0) and (0,1) (see details in the Methods section). In Fig. 4c, we visualize only *I*_(1,0)_ and observe a maximum in the region where the edge is horizontal, which vanishes smoothly as the edge becomes vertical. In Fig. 4d, we visualize only *I*_(1,0)_ and observe that the intensity is maximum at both sides and vanishes as the edges become horizontal.

Figure 4e–h shows images of the ETH Zurich logo. The letters are 15 mm width (*λ*/3), and the separation between letters varies between 10 mm (*λ*/4.4) and 15 mm. We note that this situation is considerably more challenging than the previous cases, since the object contains a much larger amount of subwavelength information. The full dynamic range image, Fig. 4e, shows intensity maxima coinciding with the edges of the letters. By reducing the dynamic range to one half, Fig. 4f, the resulting image reveals most of the features of the object, except for the lower stem of letter ‘T'. The edges of letters ‘E' and ‘H' appear clearly in the image, as well as the upper part of letter ‘T'. Figure 4g,h shows the intensities *I*_(1,0)_ and *I*_(0,1)_, which allow visualizing horizontal and vertical edges, respectively. These figures demonstrate that directional edge detection is also possible in this more complicated case.

## Discussion

We have designed and tested an acoustic metamaterial capable to transmit only information on edges and fine details of an object. These unique transmission properties stem from the combination of TRs, which enables the propagation of evanescent waves, and Bragg scattering, which avoid the transmission of propagative components. We have demonstrated the possibility to visualize edges of different objects with resolution ranging from *λ*/5 to *λ*/7.7. We foresee the ability to scale the fabrication of these devices to sizes of interest for ultrasonic imaging to improve current visualization technologies in medical and non-destructive evaluation applications. Moreover, since similar trapped modes are also found in electromagnetic waveguides (see, for example, refs 27, 28), our results may suggest the design of analogous optical edge detection devices.

## Methods

### Experimental setup

The device shown in Fig. 1b was fabricated using 3D printing. The device was placed vertically inside a box lined with 50-mm-thick absorbing foam. A 22-mm diameter loudspeaker (Clarion SRE 212H) was placed on the longitudinal axis 280 mm away from the input section. The transmitted pressure was measured with four 1/4-inch (6.35 mm) microphones (G.R.A.S. 40BD), placed flush with the inner wall, 20 mm away from the last narrow section, as indicated in Fig. 1a. The microphones were placed at the midpoint of each wall, coinciding with the nodal lines of modes (1,0) and (0,1). Hence, the transmitted amplitudes of modes (0,0), (1,0) and (0, 1) can be independently measured as *a*_(0,0)_=(*p*_1_+*p*_3_)/2=(*p*_2_+*p*_4_)/2, *a*_(0,1)_=(*p*_1_−*p*_3_)/2 and *a*_(1,0)_=(*p*_2_−*p*_4_)/2, where *p*_1_–*p*_4_ are the complex pressure measured by microphones 1–4. The pressure was measured using phase-sensitive detection to minimize noise, using a sinusoidal wave at *f*=7,740 Hz as reference signal. The metamaterial output was filled with absorbing foam to minimize backward reflections.

The visualized objects were moved in front of the waveguide input section, at 1 mm distance, using a stepper motor (Velmex). The scanning step of the 1D images in Fig. 3 was 0.6 mm (*λ*/74), and that of the 2D images in Fig. 4 was 1.6 mm (*λ*/28). The 2D images in Fig. 4 were interpolated linearly on a finer grid (0.8-mm step size) to improve the visualization resolution.

### Finite elements simulations

The simulated 1D scans shown in Fig. 3 were obtained using Comsol Multiphysics. The imaged objects and the wave-guiding structure were immersed in air and modelled as perfectly rigid. A point source was placed on the axis of the waveguide at 280 mm distance. The visualized objects were placed perpendicularly to the longitudinal axis at 1 mm distance from the input section, accordingly to experimental conditions. The propagation domain was surrounded with perfectly matched layers to simulate anechoic boundary conditions. A perfectly matched layer was also inserted in the metamaterial output to eliminate backward reflections.

## Additional information

**How to cite this article:** Molerón, M. & Daraio, C. Acoustic metamaterial for subwavelength edge detection. *Nat. Commun.* 6:8037 doi: 10.1038/ncomms9037 (2015).

## Supplementary Material

Supplementary Information Supplementary Figures 1-2 and Supplementary Notes 1-2
