# Supplementary material for: Acoustic metamaterial for subwavelength edge detection
Source: Nat Commun. 2015 Aug 25;6:8037. doi: 10.1038/ncomms9037 (PMC4560791; doi:10.1038/ncomms9037)
Supplement: Supplementary Information — Supplementary Figures 1-2 and Supplementary Notes 1-2 [file ncomms9037-s1.pdf]

SUPPLEMENTARY FIGURES

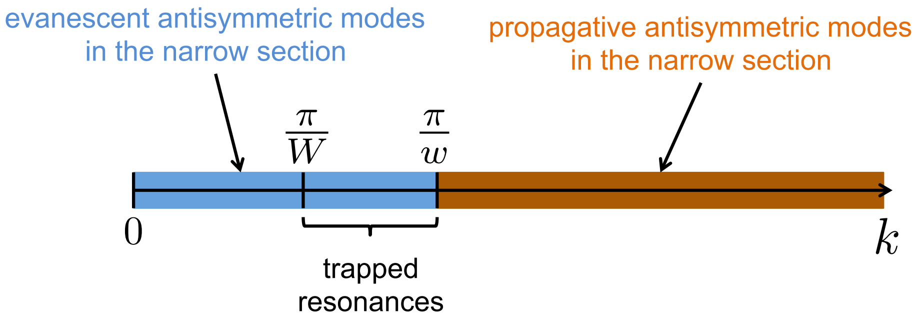

Supplementary Figure 1. Origin of the trapped resonances. The cutoff frequency of the first antisymmetric mode in the narrow section is $f = c_0/2w$ (or $k = \pi/w$) and that in the wide section is $f = c_0/2W$ (or $k = \pi/W$). Trapped resonances can appear in the band $k \in [\pi/W, \pi/w[$.

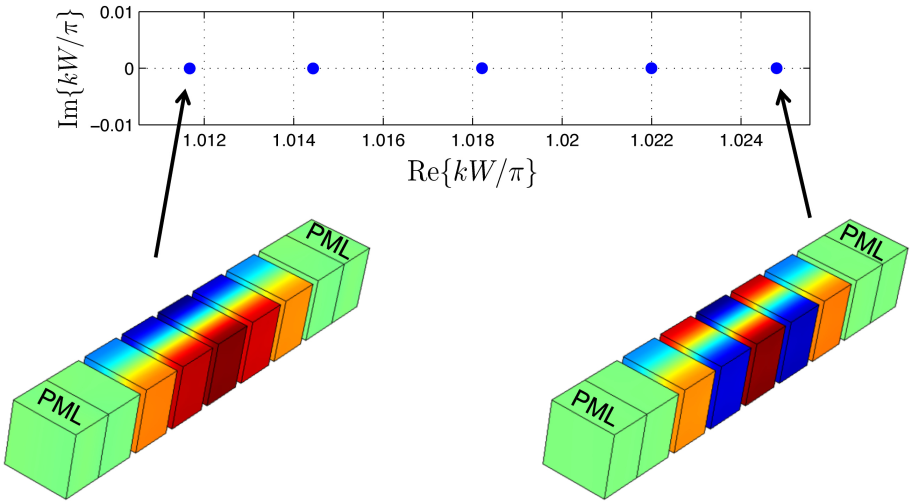

Supplementary Figure 2. Finite elements simulation of the trapped resonances. Position of the first five trapped resonances in the complex $k-$plane. Images below display the fields corresponding to the first and fifth resonances. These resonances generate the sharp transmission peaks observed in Figures 2b and 2c.

SUPPLEMENTARY NOTES

Supplementary Note 1. Origin of the trapped resonances

In this section, we briefly describe the physical mechanism leading to the formation of trapped resonances in the metatamterial shown in Figure 1a. Changes in the cross-section generate a modal coupling between modes propagating on both sides of the discontinuity. Due to the symmetry of the cross-section in y and z, symmetric modes on one side cannot couple to antisymmetric modes on the other side (their inner product vanishes). On the other hand, the cutoff frequency of the first antisymmetric mode in the wide segments, $f = c_0/2W$, is below the cutoff frequency of the same mode in the narrow segments, $f = c_0/2w$. If this mode is excited in the wide section at a frequency between $f = c_0/2W$ and $f = c_0/2/w$, it cannot couple to any propagative mode in the narrow section, and therefore it remains trapped. This situation is illustrated in the Supplementary Figure 1.

The TRs can be computed using finite elements by calculating the eigenmodes of the structure with perfectly matched layers (PML) on both extremities. Supplementary Figure 2 shows the first five TRs in the complex $k-$plane. These resonances are very close to the cutoff frequency of the first antisymmetric mode, $f = c_0/2W$ (or $k = \pi/W$), and generate the five sharp peaks observed in the transmission coefficients of Figures 2b and 2c. Their resonance frequencies are 7733 Hz, 7754 Hz, 7783 Hz, 7812 Hz and 7834 Hz, very close to the peaks observed in Figures 2b and 2c.

Supplementary Note 2. Computation of the transmission matrix, T

The pressure field p in the metamaterial is the solution to the following problem (time dependence e$^{-\jmath\omega t}$ is omitted):

$$\begin{cases} (\nabla^2 + k^2)p(x,y,z) = 0, \ \forall(x,y) \in \Omega, \\ \partial_n p(x,y,z) = 0, \ \forall(x,y) \in \partial\Omega, \end{cases} \tag{1}$$

where $k = \omega/c_0$ is the wavenumber, Ω is the air volume inside the metamaterial, $\partial\Omega$ represent perfectly rigid boundaries and ∂_n denotes the normal derivative with respect to the boundaries.

The pressure field is developed on the basis of eigenmodes of the corresponding transverse section as

$$p(x, y, z) = \sum_{m,n=1}^{\infty} \left(A_{(m,n)} \mathrm{e}^{\jmath\beta_{(m,n)}x} + B_{(m,n)} \mathrm{e}^{-\jmath\beta_{(m,n)}x}\right) \phi_{(m,n)}(y, z), \tag{2}$$

with $A_{(m,n)}$ and $B_{(m,n)}$ the amplitude of the forward and backward modes, respectively, $\beta_{(m,n)} = (k^2 - \alpha^2_{(m,n)})^{1/2}$ the longitudinal wavenumbers, where $\alpha_{(m,n)} = \left[(m\pi/w)^2 + (n\pi/w)^2\right]^{1/2}$ in the narrow section and $\alpha_{(m,n)} = \left[(m\pi/W)^2 + (n\pi/W)^2\right]^{1/2}$ in the wide section are the traverse wavenumbers. The eigenfunctions $\phi_{(m,n)}(y, z)$ are

$$\phi_{(m,n)}(y, z) = \frac{1}{w}\sqrt{(2 - \delta_{m0})(2 - \delta_{n0})} \cos\left[\frac{m\pi}{w}\left(y - \frac{w}{2}\right)\right] \cos\left[\frac{n\pi}{w}\left(z - \frac{w}{2}\right)\right] \tag{3}$$

in the narrow section and

$$\phi_{(m,n)}(y, z) = \frac{1}{W}\sqrt{(2 - \delta_{m0})(2 - \delta_{n0})} \cos\left[\frac{m\pi}{W}\left(y - \frac{W}{2}\right)\right] \cos\left[\frac{n\pi}{W}\left(z - \frac{W}{2}\right)\right] \tag{4}$$

in the wide section, where δ is the Kroneker symbol.

The scattering matrix of each element (straight segments and discontinuities) is given by

$$\mathbf{S}_i = \begin{bmatrix} \mathbf{T}_i & \mathbf{R}'_i \\ \mathbf{R}_i & \mathbf{T}'_i \end{bmatrix}, \tag{5}$$

where $i = 1, 2, \ldots, I$, with $I = 13$ the number of scattering elements, $\mathbf{R}_i$ and $\mathbf{T}_i$ are the reflection and transmission matrices for right–going incident waves, and $\mathbf{R}'_i$ and $\mathbf{T}'_i$ are the reflection and transmission matrices for left–going incident waves.

The scattering matrices of the straight segments (i = odd) are given by

$$\mathbf{S}_i = \begin{bmatrix} \mathbf{E} & [0] \\ [0] & \mathbf{E} \end{bmatrix}, \tag{6}$$

where $\mathbf{E}$ is a diagonal matrix containing the terms $\mathrm{e}^{\jmath\beta_{(m,n)}L_s}$, with L_s the length of the segment, and $[0]$ is the zero matrix. The scattering matrices of the discontinuities (i = even) are calculated from the continuity equations of pressure and normal velocity, given by

$$\begin{cases} p^{(l)} &= p^{(r)}, \\ \partial_x p^{(l)} &= \partial_x p^{(r)}, \end{cases} \tag{7}$$

where superscripts (l) and (r) indicate quantities on the left and on the right of the discontinuity, respectively. Inserting Equation (2) into Equations (7) it is possible to obtain the scattering matrix for a sudden expansion as

$$\mathbf{S}_i = \begin{bmatrix} \mathbf{T}_a & \mathbf{R}_b \\ \mathbf{R}_a & \mathbf{T}_b \end{bmatrix} \tag{8}$$

and the scattering matrix for a sudden narrowing as

$$\mathbf{S}_i = \begin{bmatrix} \mathbf{T}_b & \mathbf{R}_a \\ \mathbf{R}_b & \mathbf{T}_a \end{bmatrix}, \tag{9}$$

with

$$\begin{aligned}
\mathbf{R}_a &= \left[\mathbf{I} + \mathbf{F}\left(\mathbf{Y}^{(r)}\right)^{-1}\left({}^t\mathbf{F}\mathbf{Y}^{(l)}\right)\right]^{-1}\left[\mathbf{F}\left(\mathbf{Y}^{(r)}\right)^{-1}\left({}^t\mathbf{F}\mathbf{Y}^{(l)}\right) - \mathbf{I}\right], \\
\mathbf{T}_a &= \left(\mathbf{Y}^{(r)}\right)^{-1} {}^t\mathbf{F}\mathbf{Y}^{(l)}\left(\mathbf{I} - \mathbf{R}_a\right), \\
\mathbf{R}_b &= \left(\mathbf{Y}^{(r)} + {}^t\mathbf{F}\mathbf{Y}^{(l)}\mathbf{F}\right)^{-1}\left(\mathbf{Y}^{(r)} - {}^t\mathbf{F}\mathbf{Y}^{(l)}\mathbf{F}\right), \\
\mathbf{T}_b &= \mathbf{F}\left(\mathbf{I} + \mathbf{R}_b\right),
\end{aligned}$$

where $\mathbf{I}$ is the identity matrix, $\mathbf{F}$ is the matching matrix, containing the inner product between the modal basis, $\langle \phi^{(r)}_{(m,n)}, \phi^{(l)}_{(l,k)} \rangle = \int_s \phi^{(r)}_{(m,n)} \phi^{(l)}_{(l,k)} \mathrm{d}y\mathrm{d}z$, $\mathbf{Y}$ is a diagonal matrix containing the admittance terms $\jmath\beta_{(m,n)}$, and ${}^t\mathbf{F}$ is the transpose of $\mathbf{F}$.

Defining the matrix operator $\star$ allowing assembling two scattering matrices, $\mathbf{S}_i$ and $\mathbf{S}_{i+1}$, as

$$\mathbf{S}_i \star \mathbf{S}_{i+1} = \begin{bmatrix} \mathbf{T}_{i+1}(\mathbf{I} - \mathbf{R}'_i \mathbf{R}_{i+1})^{-1} \mathbf{T}_i & \mathbf{R}'_{i+1} + \mathbf{T}_{i+1}(1 - \mathbf{R}'_i \mathbf{R}_{i+1})^{-1} \mathbf{R}'_i \mathbf{T}'_{i+1} \\ \mathbf{R}_i + \mathbf{T}'_i(\mathbf{I} - \mathbf{R}_{i+1}\mathbf{R}'_i)^{-1} \mathbf{R}_{i+1} \mathbf{R}'_i & \mathbf{T}'_i(\mathbf{I} - \mathbf{R}_{i+1}\mathbf{R}'_i)^{-1} \mathbf{T}'_{i+1} \end{bmatrix}. \quad (10)$$

The global scattering matrix, $\mathbf{S}$, is calculated by assembling consecutively the scattering matrices of each element as

$$\mathbf{S} = \begin{bmatrix} \mathbf{R} & \mathbf{T} \\ \mathbf{T} & \mathbf{R} \end{bmatrix} = \mathbf{S}_1 \star \mathbf{S}_2 \star \cdots \star \mathbf{S}_I, \quad (11)$$

from which we extract the transmission matrix, $\mathbf{T}$.
